# Mapping the terminology of the early rescue chain to the Foundation of ICD-11: Registered report protocol

**DOI:** 10.1371/journal.pone.0350733

**Published:** 2026-06-16

**Authors:** Masresha Derese Tegegne, Samson W. Tu, Islam Ibrahim, Ronald Cornet, Sharareh Rostam Niakan Kalhori, Thomas M. Deserno

**Affiliations:** 1 Peter L. Reichertz Institute for Medical Informatics of TU Braunschweig and Hannover Medical School, Braunschweig, Germany; 2 Department of Medicine, Stanford University Medical School, Stanford, United States of America; 3 School of Population Health, Faculty of Medicine and Health, University of New South Wales, Sydney, Australia; 4 Department of Medical Informatics, Amsterdam University Medical Center, University of Amsterdam, Amsterdam, Netherlands; Ministry of Health, Sri Lanka, SRI LANKA

## Abstract

**Background:**

The World Health Organization (WHO) emphasizes digital technologies as a key accelerator for strengthening health emergency preparedness and response. One such application is the early rescue chain (ERC), which coordinates automated alerting, responding, and clinical systems. However, current ERC implementations primarily rely on spoken language and lack standardized terminology. The ERC Terminology (ERC_T) defines the minimum set of concepts required for automated and standardized ERC information exchange. The International Classification of Diseases, 11th Revision (ICD-11) extensively covers adverse events in its sections: “External causes of morbidity or mortality” and “Dimensions of external causes”. However, there is no comprehensive mapping of ERC_T to the ICD-11 Foundation (ICD-11_F), the terminological resource from which the successor to ICD-10, ICD-11 Mortality and Morbidity Statistics (MMS), is derived. Therefore, we aim to: (I) assess semantic relationships between ERC_T and ICD-11_F; (II) evaluate the overall mapping quality; and (III) identify missing concepts and semantic gaps between ERC_T and ICD-11_F.

**Methods:**

We will select ICD-11_F as the target of the mapping because of its greater specificity and its structure as a terminology rather than a classification that is constrained to a single-parent monohierarchy with residual categories. We will systematically map concepts from ERC_T to ICD-11_F using predefined mapping criteria and procedures. The mapping process addresses two complementary aspects of semantic alignment. First, we will classify the semantic relationship between an ERC_T concept and an ICD-11_F entity using HL7 FHIR ConceptMap relationships. Second, we will evaluate the quality of each mapping using ISO 21564:2025 (MapQual) measures, which provide a structured assessment of how well the ICD-11_F entity represents the ERC_T concept. Two coders will independently conduct the mapping following predefined mapping procedures. We will assess intercoder agreement using percentage and Krippendorff’s alpha (α), and will resolve disagreements through structured consensus or, if unresolved, by a senior expert.

## 1. Introduction

The Seventy-eighth World Health Assembly (WHA78) approved the extension of the global strategy on digital health to 2027 [1]. Under the Thirteenth General Program of Work (GPW13, 2019–2025) [2], the World Health Organization (WHO) strategically strengthened the architecture of health emergency preparedness and response using digital technologies as key accelerators.

A practical implementation of this digital and data-driven approach is Accident & Emergency Informatics (A&EI) [3], an interdisciplinary field that collects, analyses, and communicates data on health emergencies and disasters to support prompt decision-making and to save lives. A core component of A&EI is the early rescue chain (ERC), which coordinates the automated communication among alerting, responding, and clinical systems [4]. However, current ERCs use spoken language for communication, and a significant barrier is the lack of semantic interoperability [5].

**Fig 1:**
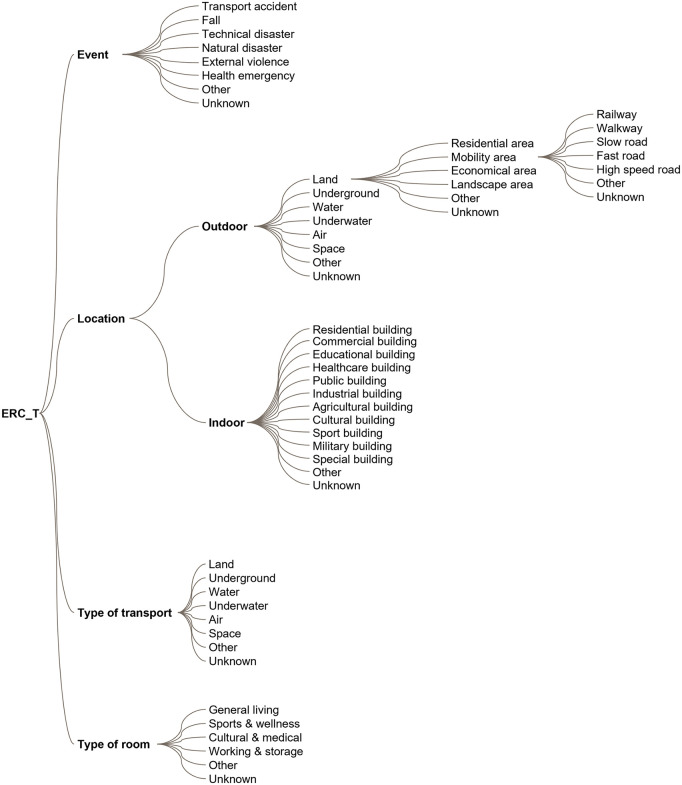
Hierarchy of concepts in ERC_T [7], which comprises five categories and 307 multi-level concepts eligible for mapping. The figure shows examples of intermediate and terminal concepts under “Land”.

Deserno & Jakob [6] emphasized the need for a standardized terminology to automate the ERC. Accordingly, we developed a minimum set of concepts to support automated information exchange in the ERC, referred to as ERC Terminology (ERC_T) [7]. ERC_T follows a mono-hierarchical, multi-axial terminology structure comprising five categories: event, outdoor location, indoor location, type of transport, and type of room (Fig 1). Each category contains a defined concept organized in a multi-level hierarchy, comprising a total of 307 ERC_T concepts, excluding residual concepts (i.e., “other” and “unknown”). The terminology explicitly defines inclusion and exclusion criteria for each concept.

The International Classification of Diseases, 11th Revision (ICD-11) covers adverse events through its dedicated chapter on “External causes of morbidity or mortality” and related Extension code under “Dimensions of external causes” [8]. The informatics architecture of ICD-11 is based on the ICD-11 Foundation (ICD-11_F), a polyhierarchical, multi-level semantic network that comprises all ICD-related content [9]. The ICD-11_F represents each clinical concept as a distinct entity, identified by a unique Uniform Resource Identifier (URI). It is part of the WHO Family of International Classifications (WHO-FIC) Foundation, which comprises over 100,000 entities across ICD, the International Classification of Functioning, Disability and Health (ICF), and the International Classification of Health Interventions (ICHI) [10]. The ICD-11 Mortality and Morbidity Statistics (ICD-11 MMS) is the main linearization of the ICD-11_F and constitutes a selected subset that meets the requirements of a statistical classification. It offers around 17,000 unique codes for diagnoses and causes of death within a fully digital and flexible structure [11]. These codes are supported by a rich set of index terms and synonyms, enabling detailed clinical expression. Accordingly, ICD-11_F contains detailed clinical entities, while residual categories such as “Other specified” and “Unspecified” are features of the ICD-11 MMS and do not exist in ICD-11_F.

Despite the availability of ERC_T and ICD-11_F, the lack of a systematic and objective assessment of their alignment hinders efforts to leverage ICD-11 to support semantic interoperability for automated emergency response. Therefore, we aim to analyze the semantic relationship and mapping quality between ERC_T and the ICD-11_F in describing adverse events.

Assessing mapping between terminologies has been extensively studied. Several studies employed the Fast Healthcare Interoperability Resources (FHIR) ConceptMap resource [12] to assess semantic relationships between terminological systems. Ohlsen et al. [13] developed algorithms that automatically generate machine-readable FHIR ConceptMap relationships from the WHO ICD-10-to-ICD-11 mapping tables to support automated mapping and the transition to ICD-11. Similarly, we conducted an exploratory manual mapping of ERC_T to ICD-11 MMS [14], applying high-level FHIR ConceptMap relationships [15]. Together, these studies demonstrate that FHIR ConceptMap provides a practical framework for implementing computable terminology mappings, thereby enabling semantic interoperability and data exchange.

In addition, several recent studies have applied ISO/TS 21564:2019 Health informatics terminology resource map quality measures (MapQual) to evaluate semantic interoperability in clinical and research datasets. Rinaldi et al. [16] used this standard to assess semantic interoperability between the International Severe Acute Respiratory and Emerging Infection Consortium (ISARIC) freestanding follow-up survey and a large multicohort COVID-19 research dataset. Vorisek et al. [17] applied the same framework to evaluate the suitability of Systematized Nomenclature of Medicine Clinical Terms (SNOMED CT) for encoding the German Corona Consensus Dataset (GECCO). Extending this approach to social and diversity data, Muzoora et al. [18] used the standard to assess the alignment of SNOMED CT and Logical Observation Identifiers Names and Codes (LOINC) with the Diversity Minimal Item Set (DiMIS). Overall, across these studies, researchers applied ISO/TS 21564:2019 equivalence assessment scores and calculated weighted-average scores to evaluate overall semantic equivalence, thereby providing a standardized assessment of semantic interoperability when integrating heterogeneous clinical and research datasets. However, these studies primarily focus on clinical and research datasets.

To our knowledge, no work systematically combines FHIR-based semantic relationship classification with ISO 21564 (MapQual) quality assessment. This registered report protocol specifies the procedure for reproducible mapping of ERC_T to ICD-11_F using FHIR and ISO standards, and a standardized assessment of semantic interoperability. Our objectives are to:

I. Assess semantic relationships;
II. Evaluate overall mapping quality; and
III. Identify missing concepts and semantic gaps.

## 2. Materials and methods

### 2.1. Definitions of terms

This section defines the basic terms used throughout this registered report protocol. The definitions reflect their operational use in mapping ERC_T to ICD-11_F (Table 1).

**Table 1:** Basic terms.

Term	Definition
Event	The occurrence of a natural or man-made incident that requires immediate response to protect life, prevent harm, or minimize damage to property or the environment. Examples are traffic accidents, accidental falls, natural or technical disasters, acts of violence, or health emergencies.
Early rescue chain (ERC)	A sequence of coordinated emergency systems, activated immediately after an event to provide timely assistance. It involves automatic alerting systems (AAS) to alert of events, responding systems to reach the affected site, and clinical systems to provide medical care or other necessary intervention.
Early rescue chain terminology (ERC_T)	A minimum set of concepts to support machine-to-machine (M2M) communication in the ERC.
Category	The top level of the ERC_T hierarchy, which contains concepts organized across multiple levels.
Concept	The fundamental unit of the ERC_T hierarchy, defined across multiple levels (intermediate or terminal). Each concept has its own definition, with inclusion and exclusion criteria where applicable.
ICD-11	The International Classification of Diseases, Eleventh Revision.
ICD-11 Foundation (ICD-11_F)	The terminological resource of detailed health-related concepts from which ICD-11 MMS is generated.
ICD-11 Morbidity and Mortality Statistics (ICD-11 MMS)	The disease and disorder reference classification of the WHO-FIC. It is the main linearization of the ICD-11_F, developed by selecting and arranging entities into a purpose-specific, limited-depth, single-parent hierarchy [9].
Mapping	The process of defining a relationship between concepts in the ERC_T and those in the ICD-11_F [19]
Map source	ERC_T
Map target	ICD-11_F
Semantic relationship	The relation type of source and target concepts as derived from FHIR ConceptMap, i.e., equivalence, broader, narrower, related to, and no match (Section 2.5.1) [15].
Quality score	The quantitative score of equivalence between the source and target concepts, defined according to ISO 21564:2025, i.e., [0,…,4], where 0 indicates the highest quality (Section 2.5.2) [20].
Mapping cardinality	For each concept in the map source, we will map to one map target concept (Section 2.4).

### 2.2. Study design

This study is a preregistered terminology mapping study. We systematically map concepts from ERC_T to ICD-11_F using predefined mapping criteria and procedure. The units of analysis are all ERC_T concepts eligible for mapping. We execute all our predefined mapping workflows in accordance with the approved registered report protocol.

### 2.3. Terminology sources and resources

#### 2.3.1. Sources terminology.

Our source terminology is ERC_T [7], which defines a minimum set of concepts for automatic alerts within the ERC across five categories: event, outdoor location, indoor location, type of transport, and type of room (Fig 1). Each category contains multi-level concepts with their own definitions, along with inclusion and exclusion criteria provided where applicable.

#### 2.3.2. Target terminology.

**Fig 2:**
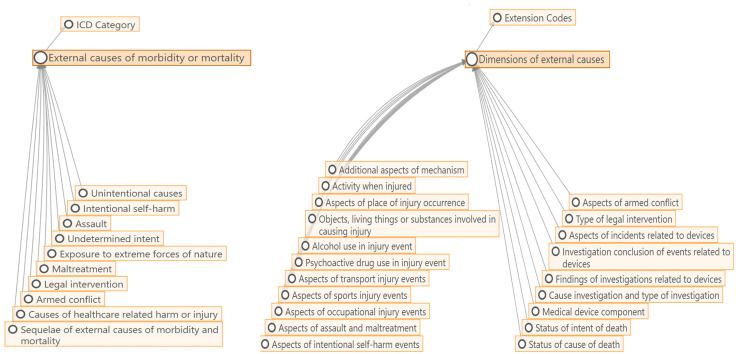
Target ICD-11_F section “External causes of morbidity or mortality” and “Dimensions of external causes” (screenshot reproduced from the WHO-FIC Maintenance Platform) [25,26].

Our target terminology is the ICD-11_F, which includes a dedicated section for the detailed description of “Events” through “External causes of morbidity or mortality” [21] and the related Extension codes under “Dimensions of external causes” [22] (Fig 2). Building on our previous work demonstrating that the ERC_T’s ICD-11 MMS map targets include below-the-shoreline ICD-11_F entities (represented in ICD-11 MMS as index terms) and ICD-11 MMS residual categories that affect semantic interoperability [14,23], we shift the target to ICD-11_F, which is structured as a terminology rather than a classification [9]. This shift avoids these constraints and supporting the direct mapping of ERC_T concepts to ICD-11_F entities. Using the ICD-11_F API (v2.5) [24], we extract approximately 14,754 unique concept URIs that represent the complete set of entities under the “External causes of morbidity or mortality” and the “Dimensions of external causes”.

### 2.4. Scope and eligibility criteria

The mapping is limited to valid ERC_T entries with explicit definitions. Residual entries such as “other” and “unknown” are excluded. Within ICD-11_F, the scope is restricted to “External causes of morbidity or mortality” and their associated dimensions in the Extension codes section, as other sections are not relevant to the ERC.

We follow a one-to-one mapping cardinality. This is because ERC_T is a hierarchical terminology of predefined concepts (e.g., a specific event, location, transport type, or type of room) rather than a collection of pre-coordinated composite terms. The target, ICD-11_F, is a terminology in which a URI uniquely identifies each entity. Therefore, single-target semantic equivalence is the appropriate approach for assessing semantic alignment between the two terminologies. Compositional cardinality scenarios (one-to-many, many-to-one, or many-to-many) lie outside the scope of this terminology mapping, as they pertain to the combined use of terminologies in real-world coding, such as encoding a complete event record.

### 2.5. Mapping framework

#### 2.5.1. Semantic relationship.

I. **Definition**

The FHIR “ConceptMap” framework [12] defines explicit semantic relationship types between source and target concepts. The relationship element is bound to a value set derived from the FHIR CodeSystem “ConceptMapRelationship” [15] (Table 2). In our work, we depart from the FHIR “ConceptMapRelationship” in two ways. First, the “Related to” relationship is defined as “The concepts are related to each other, but the exact relationship is not known.” However, the subcategories “Equivalent,” “Broader,” and “Narrower” do not cover the case where the source intersects (overlaps with) the target. Instead of adding a subcategory, we include such cases in the “Related to” category. Second, the FHIR “ConceptMapRelationship” defines the semantic relationship type “Not related to”; however, this relationship does not apply to our mapping. We consider only a predefined set of source concepts, while target concepts are identified dynamically and are not predefined.

**Table 2:** FHIR conceptMapRelationship and mapping outcome [15].

Semantic relationship/ outcome	Description
Related to	The concepts are related to each other, but the exact relationship is not known.
Equivalent	The definitions of the concepts mean the same thing.
Source is broader than target	The source concept is broader in meaning than the target concept.
Source is narrower than target	The source concept is narrower in meaning than the target concept.
Not related to^a^	This is an explicit assertion that the target concept is not related to the source concept.
No match^b^	There is no matching target concept for the source concept.

a Not applicable

b No match represents a mapping outcome rather than a semantic relationship and is therefore represented using “ConceptMap.group.unmapped” rather than “ConceptMapRelationship”.

Therefore, if we identify no suitable target concept, this indicates the absence of a mapping rather than an explicit semantic judgment of non-relatedness. We represent these cases using the “ConceptMap.group.unmapped” [27] element, as defined in the FHIR specification, and label them as “No match”.

II. **Examples**

A necessary part of a mapping framework is the criteria for operationalizing the assignment of semantic relationships. This is especially important because of the mismatch between the conceptual models of ERC_T and ICD-11_F: ERC_T focuses on describing adverse events to alert emergency services, whereas ICD-11_F is designed for classifying external causes of injury. In this section, we explore the assignment of semantic relationships with some examples.

The ERC_T concept “Transport accident” is related to the ICD-11_F entity “Unintentional transport injury event.” However, the source concept also includes transport accidents resulting from intentional or assaultive acts. Therefore, it has a broader semantic scope than the target entity “Unintentional transport injury event.”

The ERC_T concept “Pedestrian” is defined as a type of land transport referring to a person traveling on foot, including standing, walking, or running, and encompasses “Pedestrian using a mobility aid” and “Pedestrian without a mobility aid”. The ICD-11_F entity “Pedestrian as mode of transport of person injured in transport event” accurately reflects the ERC_T definition. Although ERC_T defines pedestrians as a separate land transport and models transport accidents independently, ICD-11_F explicitly describes pedestrians as a mode of transport in relation to injury. Even with this difference, both terms refer to the same transport role in traffic-related adverse events and are therefore considered equivalent.

The ERC_T concept “Technical disaster” refers to adverse events caused by direct human action. Its terminal concepts are, for instance, “Building collapse”, “Explosion”, and “Fire”. Although these concepts can be mapped based on their specific definitions, no corresponding parent-level in the ICD-11_F encompasses the full scope of “Technical disaster.” Consequently, the mapping is classified as no match (Table 3).

**Table 3:** Examples of semantic relationships.

Source: ERC_T concept (Definition)	Target: ICD-11_F entity (URL)	Semantic relationship/ outcome
Transport accident (Adverse event in transport resulting in property damage and/or bodily injury)	Unintentional transport injury event (http://id.who.int/icd/entity/1609069486)	Source is broader than target
Pedestrian (A type of land transport referring to a person traveling on foot, whether standing, walking, or running)	Pedestrian as mode of transport of person injured in transport event (http://id.who.int/icd/entity/1809796899)	Equivalent
Technical disaster (Adverse event with one or more direct human causes)	---	No match

III. Pros and cons of FHIR CodeSystem “ConceptMapRelationship.”

**Pros**

- Defines semantic relationships between concepts across terminologies [15].
- Enables interoperability and data exchange in FHIR systems [28].
- Supports automated terminology mapping through machine-processable semantic relationship types, facilitating integration across systems [13,29].
- Widely adopted and open source [30].

**Cons**

- Semantic relationships between terminology concepts are not turned into a numeric score, making it challenging to quantify the overall semantic interoperability [15].
- Less suitable for research requiring a detailed evaluation of mapping quality [31].
- Does not explicitly cover the case where the source intersects the target.
- Relies on predefined source and target concepts and represents unmatched cases as mapping outcomes rather than semantic relationships [27].

#### 2.5.2. Mapping quality.

I. Definition

ISO 21564:2025, Health Informatics — Terminology resource map quality measures (MapQual) provides a framework for quantifying the quality of maps between terminological resources [20]. The framework defines equivalence measures as the degree to which the meanings of a source and a target concept match. Mapping quality is evaluated by assigning an equivalence score to each map, with the lowest score (0) corresponding to the highest quality match (Table 4).

**Table 4:** ISO 21564:2025 (MapQual) map quality measure [20].

Quality score	Description
0	Equivalent meaning, where the highest quality equivalence is represented by the lowest number, 0 is the ‘best score’.
1	The source is wholly included in the target.
2	The source is partially included in the target.
3	Source is mapped; however, there were many options of possible concepts and overlaps. The map produced is a best comparison rather than an actual correspondence. Such a map requires significant manual input to create and maintain, and it should be used with care.
4	No map possible.

### II. Examples

We use the same examples as in Section 2.5.1.2. Mapping the ERC_T concept “Transport accident” to the ICD-11_F entity “Unintentional transport injury event” results in a partial inclusion. The source concept encompasses transport accidents of various intents (i.e., unintentional, intentional, and undetermined). Only unintentional transport accidents fall within the semantic scope of the selected ICD-11_F target entity. Therefore, the source concept can be partitioned into two disjoint subsets: one included in the target and the other excluded from it. Accordingly, this mapping is assigned a quality score of 2.

Mapping the ERC_T concept “Pedestrian” to the ICD-11_F entity “Pedestrian as mode of transport of person injured in transport event” received a quality score of 0, reflecting semantic equivalence despite differences in modeling. Furthermore, mapping the ERC_T concept “Technical disaster” to ICD-11_F receives a quality score of 4, reflecting that no valid ICD-11_F target entity adequately represents the full scope of the source concept (Table 5).

**Table 5:** Example of quality score.

Source: ERC_T concept (Definition)	Target: ICD-11_F entity (URL)	Quality score
Transport accident (Adverse event in transport resulting in property damage and/or bodily injury)	Unintentional transport injury event (http://id.who.int/icd/entity/1609069486)	2
Pedestrian (A type of land transport referring to a person travelling on foot, whether standing, walking, or running)	Pedestrian as mode of transport of person injured in transport event (http://id.who.int/icd/entity/1809796899)	0
Technical disaster (Adverse event with one or more direct human causes)	---	4

III. Pros and Cons of ISO 21564:2025

**Pros**.

- Provides quality assessment of terminology mappings [20].
- Offers numeric or graded measures of mapping quality for evaluating semantic interoperability between two terminology systems [20].
- Designed for research contexts requiring methodological rigor, enabling evaluation and comparison of overall mapping quality across datasets [20].

#### Cons.

- Evaluates mapping quality but does not specify semantic relationship types (e.g., broader, narrower, related) [20].
- Limited automation requires expert-driven and conservative semantic assessment [16].
- Newly standardized (2025) [20] and integration with FHIR requires separate guidance [32].
- Not open source [20].

#### 2.5.3. Complementarity of mapping frameworks.

Although certain mapping labels may appear conceptually aligned across the two frameworks, for instance, the “Equivalent” semantic relationship and the quality score “0”, these overlaps reflect intentional methodological complementarity rather than redundancy. The frameworks are neither intended to replace one another nor to be used interchangeably. Instead, each provides a distinct perspective on semantic relationship and mapping quality, and will be applied independently to address the separate research questions.

Therefore, our registered report protocol applies both distinct mapping frameworks to address separate objectives. HL7 FHIR ConceptMap framework establishes semantic relationships, while ISO 21564:2025 (MapQual) measures mapping quality.

### 2.6. Mapping procedure

**Fig 3:**
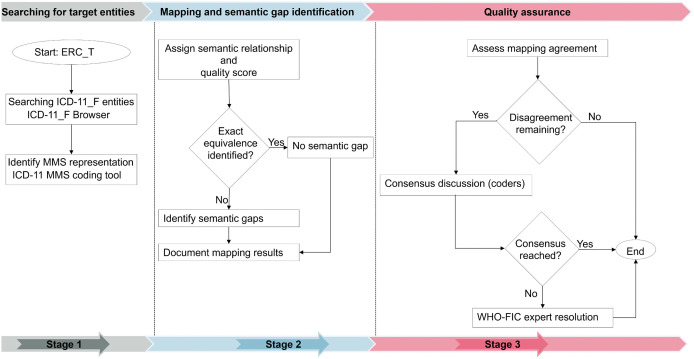
Procedure for mapping the ERC_T to ICD-11_F.

The mapping procedure follows a sequence of predefined stages (Fig 3). Each stage builds on the output of the previous stage and is applied consistently across all ERC_T concepts.

#### 2.6.1. Searching for target entities.

In the first stage, two ICD-11 coders will independently search for each ERC_T concept in the ICD-11_F using the WHO-FIC Foundation Browser [33], without access to each other’s mappings. They will not consider concepts from the ICF and ICHI included in the browser, as they are outside the scope of this registered report protocol. The ERC_T concept serves as the source. To enhance target retrievals, coders will consider the source concept’s definition, inclusion criteria, and exclusion criteria. In addition, the coders will use the ICD-11 MMS coding tool [34] to determine how the identified ICD-11_F entities are represented within ICD-11 MMS. This process will identify entities represented with a distinct ICD-11 MMS code, those listed as index terms, and those classified in a residual category.

#### 2.6.2. Mapping and semantic gap identification.

After identifying relevant ICD-11_F entities, the coders will classify the semantic relationship for each mapping using the HL7 FHIR ConceptMap framework. They will assign mapping quality scores according to ISO 21564:2025, based on the following predefined assumptions underlying mapping decisions (Section I). When mappings are not equivalent, the coders will identify semantic gaps between the ERC_T concept and the ICD-11_F entities.

I. **Assumptions underlying mapping decisions**

These assumptions define methodological decision rules for mapping and do not reflect deficiencies in either ERC_T or ICD-11_F. To ensure reproducibility, we classify mapping decisions based on the following assumptions, informed by our feasibility analysis of the target ICD-11_F entities (Section 2.7).

A. **Absence of a suitable target entity**

If no ICD-11_F target entity exists to represent the ERC_T concept, coders will assign the mapping outcome of “No match”, resulting in a quality score of “4” (No map possible). This rule operationalizes the feasibility check and supports the identification of missing ERC_T concepts in the ICD-11_F. Examples are the ERC_T concepts “Technical disaster”; locations such as “Underwater”, “Air”, and “Space”; and “Cableway” as a form of land transport.

B. **Equivalence semantic alignment**

The coders will assign the semantic relationship “Equivalent” and the quality score “0” if both concepts are semantically equivalent. However, differences in system modeling perspective, such as the event-based representation in ERC_T and classification of external causes of injury in ICD-11_F, do not, by themselves, preclude equivalence across the two frameworks, provided that they have the same semantic meaning. For instance, “Pedestrian” (event-focused) and “Pedestrian as mode of transport of person injured in transport event” (cause of injury-focused) refer to the same type of transport.

C. **Partial semantic alignment**

If a target ICD-11_F entity exists but is not semantically equivalent, the coders will assign the semantic relationship “source is broader than target,” “source is narrower than target,” or “related to” according to the HL7 FHIR ConceptMap framework and a quality score of “1–3” according to ISO 21564:2025.

Given the operational alignment between the two frameworks, the semantic relationship “Source is narrower than target” is interpreted as a quality score of “1” (The source is wholly included in the target). Similarly, the semantic relationship “Source is broader than target” is interpreted as a quality score of “2” (The source is partially included in the target). We illustrate this with the following examples:

- The ERC_T concept “Engine cycle” has the definition “Motorized vehicle without a cabin” and maps to the ICD-11_F entity “Motorized two- or three-wheeled vehicle.” The semantic relationship is classified as “Source is narrower than target,” as every engine cycle is a subset of a motorized two- or three-wheeled vehicle. Since the target ICD-11_F entity fully encompasses the ERC_T concept, the mapping is assigned a quality score of “1” (The source is wholly included in the target). The constraint “without a cabin” semantically narrows the source concept by restricting its set of instances, but the restricted set remains fully contained within the target concept and therefore does not alter the mapping decision.
- The ERC_T concept “Fall,” defined as an adverse event in which a person loses stability and descends to a lower level, is mapped to the ICD-11 target entity “Unintentional fall”. The semantic relationship is classified as “Source is broader than target,” as the ERC_T concept encompasses falls of varying intent. Accordingly, the source concept partitions into two disjoint subsets: unintentional falls within the target, and intentional or undetermined intent falls outside the target. Therefore, this mapping receives a quality score of “2” (The source is partially included in the target).

However, we cannot always interpret the two frameworks directly in relation to each other. The FHIR semantic relationship “related-to” corresponds to an ISO mapping quality score of “2” or “3”, depending on the nature of the relationship. If a “related-to” relationship indicates a well-defined intersection, we will assign a quality score of “2” (The source is partially included in the target). In contrast, if no clear intersection exists, we will assign a quality score of “3” (Source is mapped; however, there were many options of possible concepts and overlaps. The map produced is a best comparison rather than an actual correspondence. Such a map requires significant manual input to create and maintain, and it should be used with care). The following two examples illustrate this distinction:

- The ERC_T concept “Residential area,” defined as “Outdoor land area used for daily living activities,” maps to the ICD-11_F entity “Home.” The semantic relationship between the two is “Related to,” as neither concept fully subsumes the other: ERC_T concept covers all outdoor residential areas, including those that extend beyond homes (e.g., parks, sports and entertainment areas, squares, and marketplaces), whereas ICD-11_F’s “Home” is limited to a single dwelling and its indoor spaces. The two concepts share a well-defined intersection: the outdoor spaces directly attached to a single dwelling (e.g., garden, roof, backyard, garage). The resulting quality score is “2” (the source is partially included in the target).
- The ERC_T concept “Diver-assisted device,” defined as “Underwater transport devices that directly augment a diver’s capabilities,” maps to the ICD-11_F entity “Underwater diving equipment.” The semantic relationship is “Related to” because both refer to equipment used by divers underwater. However, there is no well-defined intersection, as the ERC_T concept refers to devices that actively assist a diver’s movement (e.g., propulsion devices, assist exoskeletons), whereas ICD-11_F refers to the passive gear divers wear underwater (e.g., aqualung, wetsuit, mask, fins). This results in a quality score of “3” (Source is mapped; however, there are many options of possible concepts and overlaps. The map produced is the best comparison rather than an actual correspondence. Such a map requires significant manual input to create and maintain, and should be used carefully).

II. **Documentation**

The two coders will independently perform the mappings, without access to each other’s sections, and will document their results in separate, structured spreadsheets (Excel, Microsoft Corporation, Redmond, WA, USA). For each predefined ERC_T concept, they will record the mapped ICD-11_F entity (title and Foundation URL), its representation in the ICD-11 MMS, the assigned semantic relationships, the quality scores, and any identified gaps.

#### 2.6.3 Quality assurance.

Based on this documentation, a WHO-FIC expert will conduct a pilot assessment and, where needed, provide guidance or training to address systematic issues in applying our predefined mapping procedure and ICD-11 coding principles. Then, we will calculate the percentage agreement of target entity selection and evaluate intercoder reliability using Krippendorff’s alpha (α) [35]. We will interpret inter-coder reliability using the predefined thresholds (Table 6), adapted from Krippendorff [35] and Marzi et al. [36]. In cases of disagreement, coders will resolve them through structured consensus discussions. If they cannot reach a consensus, they will refer the case to the WHO-FIC expert for final decision.

**Table 6:** Predefined thresholds for interpreting Krippendorff’s alpha (α) [35,36].

Krippendorff’s alpha (α) value	Interpretation	Action
α = 1	Perfect agreement	• Accept mapping outcomes for drawing conclusions • No disagreements to resolve
α ≥ 0.80	Satisfactory level of agreement	• Accept mapping outcomes for drawing conclusions • Resolve disagreements
0.67 ≤ α < 0.80	Tentative agreement	• Interpret mapping outcomes with caution • Resolve disagreements
α < 0.67	Poor agreement	• Re-examine mapping rules • Provide additional coder training • Resolve disagreements
α = 0	Agreement at chance level	• Re-examine mapping rules • Provide additional coder training • Re-code all mappings • Resolve disagreements
α < 0	Systematic disagreement	• Re-examine mapping rules • Provide additional coder training • Re-code all mappings • Resolve disagreements

### 2.7. Feasibility of target entities

This section reports a preliminary, single-coder feasibility analysis conducted to determine whether target ICD-11_F entities exist for the ERC_T concepts, without assessing semantic relationships and quality score. We limit the assessment to confirming the availability of potentially relevant target ICD-11_F entities to support the proposed registered report protocol, and it does not constitute a final mapping decision or validated mapping results.

**Fig 4:**
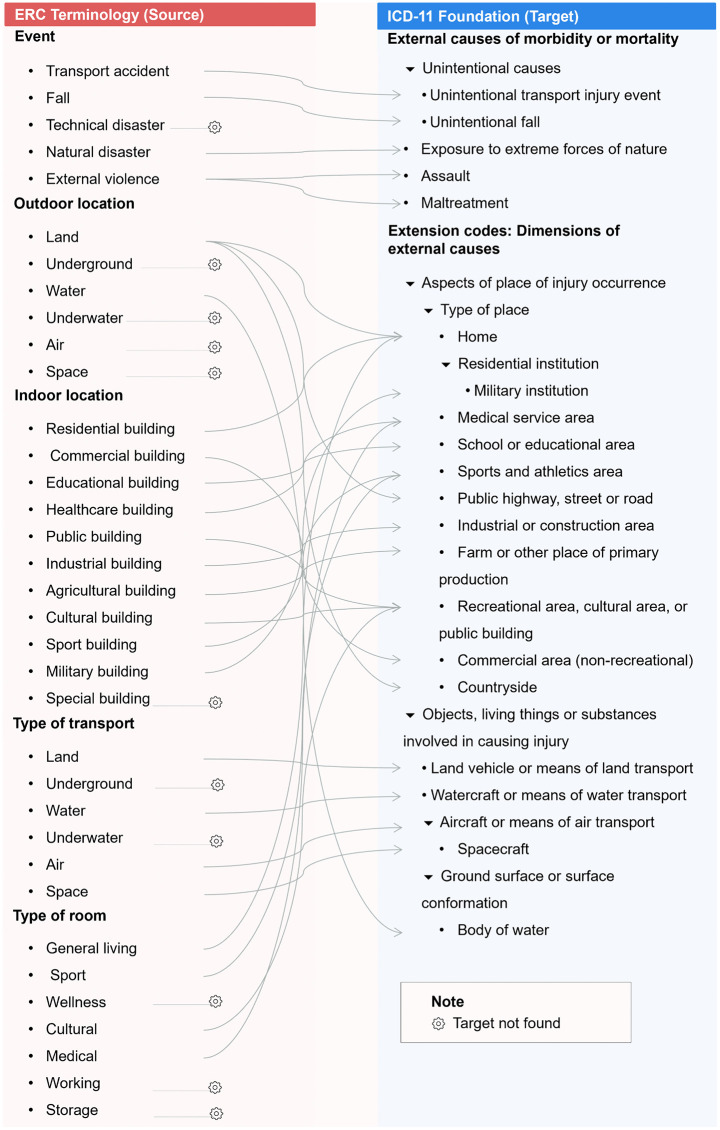
Feasibility analysis of target ICD-11_F entities for ERC_T concepts. Single-coder example mapping illustrating only potential targets, without assignment of semantic relationships or quality scores.

At the ERC_T category level, the ICD-11_F section “External causes of morbidity or mortality” constitutes a potential target for the ERC_T event category. The ICD-11_F extension code section “Dimensions of external causes” is a potential target for the remaining ERC_T categories: indoor and outdoor locations, types of transport, and types of rooms. Within this structure, we group outdoor and indoor locations, as well as room types, under the target ICD-11_F entity “Aspects of place of injury occurrence.” We group transport types under the target ICD-11_F entity “Objects, living things or substances involved in causing injury” (Fig 4). As an illustrative example of the feasibility assessment, we focus on the ERC_T concepts within the “Event” category. The ICD-11_F entities “Unintentional transport injury event” and “Unintentional fall” represent potential target entities for the ERC_T concepts “Transport accident” and “Fall,” because the term “unintentional” closely aligns with “accidental.” However, ERC_T does not encode intent, whereas intent is a central organizing principle in the ICD-11_F section “External causes of morbidity or mortality.” For example, a transport accident in ERC_T does not distinguish between intentional and unintentional events, while ICD-11_F explicitly differentiates between these intent options. These conceptual differences define important constraints for the subsequent mapping process and influence the assignment of the semantic relationship and quality score.

The ICD-11_F entity “Exposure to extreme forces of nature” represents a potential target for the ERC_T concept “Natural disaster.” In addition, ERC_T concepts such as “Technical disaster” and “External violence” do not correspond to a single consolidated entity or correspond to two target ICD-11_F entities. Although we identify two target ICD-11_F entities during the feasibility assessment (e.g., external violence), we prioritize and select a single target entity in accordance with the predefined one-to-one mapping procedure (Fig 4).

## 3. Discussion

### Anticipated outcomes

The first anticipated outcome of this registered report protocol is the systematic assessment of the semantic relationship between each ERC_T concept and ICD-11_F entities using a predefined HL7 FHIR ConceptMap relationship. We will identify the semantic relationship for each ERC_T-to-ICD-11_F mapping. This establishes a logic that can be easily translated into a machine-processable format, thereby supporting interoperability and quality data exchange in a future FHIR-compliant environment.

The second anticipated outcome is to evaluate mapping quality using the ISO 21564:2025 (MapQual) equivalence assessment score. We will assign a quality score from (0–4) for each ERC_T–ICD-11_F mapping, and calculate a weighted average to summarize the overall mapping quality. This approach enables quantification of the overall semantic alignment between ERC_T and ICD-11_F and facilitates comparison with mapping quality results reported in other ISO-based studies. Lower weighted average scores indicate stronger semantic alignment, while higher scores reflect greater reliance on best-comparison mappings.

Finally, based on the semantic relationships and mapping quality, we will identify the missing ERC_T concepts and characterize semantic gaps between ERC_T and ICD-11_F. These findings will inform future refinement of ERC_T and the proposal of potential enhancements to ICD-11 content, thereby supporting semantic interoperability between ERC and ICD-11. Furthermore, this registered report protocol provides a foundation for future research. Such research may include developing algorithms to automate the mapping process and extending the methodology to other terminologies and classifications in health informatics.

### Anticipated limitations

This report protocol has several anticipated limitations. Although “Health emergency” exists as an intermediate concept under the ERC_T event category, we do not operationalize it into a structured set of concepts with inclusion and exclusion criteria suitable for terminology mapping. Consequently, we do not map health emergency events in this registered report protocol. Therefore, interpretations of future findings apply only to the ERC_T concepts explicitly operationalized by this registered report protocol.

We deliberately restricted the scope to the ICD-11_F sections “External causes of morbidity or mortality” and the related extension codes under “Dimensions of external causes,” following a detailed review of the current ERC_T concepts against the ICD-11_F. However, other ICD-11_F sections may capture additional aspects of emergency events, injury mechanisms, or contextual response settings. Extending the ERC_T concepts to cover such entities lies beyond the aim of this registered report protocol and will be considered in future work.

Although the source terminology is a nomenclature, ICD-11_F is semantically rich and may contain several plausible target entities for a single source concept. In these cases, the coder must select the most appropriate single target, a subjective process that may degrade mapping quality. There is also the possibility of coder subjectivity in semantic interpretation. We will mitigate this by applying predefined mapping decision rules and mapping examples. We will further diagnose coder subjectivity through inter-coder reliability analysis (Section 2.6.3) and resolve it through structured consensus discussions. Furthermore, the findings of this study are specific to the terminology mapping between ERC_T and ICD-11_F within the defined scope, and cannot be directly generalized to other emergency informatics contexts.
