# Supplementary material for: Mapping the terminology of the early rescue chain to the Foundation of ICD-11: Registered report protocol
Source: PLoS One. 2026 Jun 16;21(6):e0350733. doi: 10.1371/journal.pone.0350733 (PMC13271442; doi:10.1371/journal.pone.0350733)
Supplement: Attachment — Submitted filename: Response to Reviewers.docx [file pone.0350733.s001.docx]

**Response letter for "[PONE-D-26-10568]: Mapping the terminology of the early rescue chain to the Foundation of ICD-11: Registered report protocol."**

Dear Editor and Reviewers,

We thank the editorial team and reviewers for their time, expertise, and constructive feedback.

We have prepared a point-by-point response addressing each journal requirement and reviewer suggestion. For each point, we first restate the comment, followed by our detailed response. Amendments are clearly marked in the revised manuscript using track changes.

In addition, we hereby confirm that the research we have described in our manuscript, including participant recruitment, data collection, modification, or processing, has not started and will not start until after our paper has been accepted to the journal PLOS One. Furthermore, for our study, we do not need to collect data or recruit participants.

Please let us know if any further changes or clarifications are needed.

Best regards,

The Authors

------------------------------------------------------

**Journal Requirements (JR)**

-------------------------------------------------------

When submitting your revision, we need you to address these additional requirements.

**Comment_JR#1:** Please ensure that your manuscript meets PLOS ONE's style requirements, including those for file naming. The PLOS ONE style templates can be found at <https://journals.plos.org/plosone/s/file?id=wjVg/PLOSOne_formatting_sample_main_body.pdf> and <https://journals.plos.org/plosone/s/file?id=ba62/PLOSOne_formatting_sample_title_authors_affiliations.pdf>

**Response_JR#1:** We confirm that we have prepared the manuscript in accordance with PLOS ONE's style requirements, including file naming conventions.

**Comment_JR#2:** In your cover letter, please confirm that the research you have described in your manuscript, including participant recruitment, data collection, modification, or processing, has not started and will not start until after your paper has been accepted to the journal (assuming data need to be collected or participants recruited specifically for your study). In order to proceed with your submission, you must provide confirmation.

**Response_JR#2:** We have revised the confirmation statement in our cover letter to address this requirement, as follows:

We hereby confirm that the research we have described in our manuscript, including participant recruitment, data collection, modification, or processing, has not started and will not start until after our paper has been accepted to the journal PLOS One. Furthermore, for our study, we do not need to collect data or recruit participants.

**Comment_JR#3:** Please note that PLOS One has specific guidelines on code sharing for submissions in which author-generated code underpins the findings in the manuscript. In these cases, we expect all author-generated code to be made available without restrictions upon publication of the work. Please review our guidelines at https://journals.plos.org/plosone/s/materials-and-software-sharing#loc-sharing-code and ensure that your code is shared in a way that follows best practice and facilitates reproducibility and reuse.

**Response_JR#3:** We confirm that no author-generated code has been developed at this stage. The planned analyses are descriptive in nature and will be performed using standard statistical software.

**Comment_JR#4:** If the reviewer comments include a recommendation to cite specific previously published works, please review and evaluate these publications to determine whether they are relevant and should be cited. There is no requirement to cite these works unless the editor has indicated otherwise.

**Response_JR#4:** As the reviewers did not recommend specific citations, no additional references have been included.

**Comment_JR#5:** Please review your reference list to ensure that it is complete and correct. If you have cited papers that have been retracted, please include the rationale for doing so in the manuscript text, or remove these references and replace them with relevant current references. Any changes to the reference list should be mentioned in the rebuttal letter that accompanies your revised manuscript. If you need to cite a retracted article, indicate the article’s retracted status in the References list and also include a citation and full reference for the retraction notice.

**Response_JR#5:** We have carefully reviewed our reference list to ensure completeness and accuracy. We do not cite any retracted articles in the manuscript.

------------------------------------------------------

**Reviewer #1 (R1)**

I. **General comments**

-------------------------------------------------------

This well-designed, timely registered report protocol addresses an important issue in emergency health informatics and semantic interoperability. The manuscript has several strengths, including strong academic rationale, use of internationally accepted standards, rigorous mapping methodology, independent coder-based validation, and appropriate reliability assessment. The planned study is likely to make a meaningful contribution to the interoperability of emergency response systems and the use of ICD-11 in automated health information exchange.

**Response:** We appreciate your positive assessment of our registered report protocol. We have thoroughly addressed all subsequent comments below to further enhance the manuscript.

II. **Specific comments**

-------------------------------------------------------
**Comment_R1#1**: Provide stronger justification for the one-to-one mapping cardinality.

The protocol specifies a one-to-one mapping approach, whereby each ERC_T concept is mapped to a single ICD-11_F target concept. While this simplifies the mapping framework and may facilitate analysis, the rationale for restricting the study to this cardinality should be more explicitly justified.

In terminology mapping studies, especially when working with rich semantic resources such as the ICD-11 Foundation, concepts may sometimes be more accurately represented through one-to-many mappings, post-coordination, etc. Restricting the process to a single target concept may underestimate the true semantic alignment and could potentially inflate the number of “no match” or low-quality mappings. The authors are encouraged to provide a clear justification for this design choice and briefly discuss how this restriction may influence mapping outcomes.

**Response_R1#1: Agree**. We provided an explicit justification for the one-to-one cardinality at the end of Section 2.4 (Scope and eligibility criteria), which reads as follows:

We follow a one-to-one mapping cardinality. This is because ERC_T is a hierarchical terminology of predefined concepts (e.g., a specific event, location, transport type, or type of room) rather than a collection of pre-coordinated composite terms. The target, ICD-11_F, is a terminology in which a URI uniquely identifies each entity. Therefore, single-target semantic equivalence is the appropriate approach for assessing semantic alignment between the two terminologies. Compositional cardinality scenarios (one-to-many, many-to-one, or many-to-many) lie outside the scope of this terminology mapping, as they pertain to the combined use of terminologies in real-world coding, such as encoding a complete event record.

In addition, we have acknowledged how this restriction may influence mapping outcomes in the Anticipated Limitations section, which reads as follows:

Although the source terminology is a nomenclature, ICD-11_F is semantically rich and may contain several plausible target entities for a single source concept. In these cases, the coder must select the most appropriate single target, a subjective process that may degrade mapping quality.

We also note that our mitigation measures to manage coder subjectivity are described in "Response_R1#4c".

**Comment_R1#2:** Clarify the decision rules for ambiguous or partially overlapping concepts:

The protocol explains how to use HL7 FHIR ConceptMap relationships and ISO 21564 quality scores, however it should go into more depth about how to handle ambiguous ideas, partially overlapping concepts, and numerous equally plausible targets. I suggest including one or two additional worked examples of “related-to” mappings, particularly to illustrate how coders should distinguish between quality scores 2 and 3 in borderline cases where semantic overlap is not immediately obvious. Providing clearer operational rules or additional worked examples would improve reproducibility and reduce subjective variation between coders.

**Response_R1#2: Agree**. We clarified our decision rules by adding two examples that illustrate how coders should distinguish between quality scores "2" and "3" for "related-to" mappings, as follows:

**Example 1 (Quality score 2: "Related to" with a well-defined intersection):**

The ERC_T concept "Residential area," defined as "Outdoor land area used for daily living activities," maps to the ICD-11_F entity "Home." The semantic relationship between the two is "Related to," as neither concept fully subsumes the other: ERC_T concept covers all outdoor residential areas, including those that extend beyond homes (e.g., parks, sports and entertainment areas, squares, and marketplaces), whereas ICD-11_F's "Home" is limited to a single dwelling and its indoor spaces. The two concepts share a well-defined intersection: the outdoor spaces directly attached to a single dwelling (e.g., garden, roof, backyard, garage). The resulting quality score is "2" (the source is partially included in the target).

**Example 2 (Quality score 3: "Related to" without a well-defined intersection):**

The ERC_T concept "Diver-assisted device," defined as "Underwater transport devices that directly augment a diver's capabilities," maps to the ICD-11_F entity "Underwater diving equipment." The semantic relationship is "Related to" because both refer to equipment used by divers underwater. However, there is no well-defined intersection, as the ERC_T concept refers to devices that actively assist a diver's movement (e.g., propulsion devices, assist exoskeletons), whereas ICD-11_F refers to the passive gear divers wear underwater (e.g., aqualung, wetsuit, mask, fins). This results in a quality score of "3" (Source is mapped; however, there are many options of possible concepts and overlaps. The map produced is the best comparison rather than an actual correspondence. Such a map requires significant manual input to create and maintain, and should be used carefully).

**Comment_R1#3:** Include predefined thresholds for Krippendorff’s alpha interpretation:

The use of Krippendorff’s alpha is appropriate and strengthens the methodological rigor of the study. However, the protocol would benefit from including predefined thresholds for interpretation. Including such thresholds is particularly important in a registered report protocol, as it reduces analytical flexibility and improves transparency in how intercoder reliability will be interpreted.

**Response_R1#3: Agree**. We added a new table (Table 6) in Section 2.6.3 (Quality assurance), presenting predefined thresholds for Krippendorff's alpha (α) interpretation, adapted from Krippendorff [35] and Marzi et al. [36], along with the recommended action at each threshold. The added section reads as follows:

We will calculate the percentage agreement of target entity selection and evaluate intercoder reliability using Krippendorff's alpha (α) [35]. We will interpret inter-coder reliability using the predefined thresholds (Table 6), adapted from Krippendorff [35] and Marzi et al. [36]. In cases of disagreement, coders will resolve them through structured consensus discussions. If they cannot reach a consensus, they will refer the case to the WHO-FIC expert for final decision.

**Table 6. Predefined thresholds for interpreting Krippendorff's alpha (α) [35,36].**

| **Krippendorff's alpha (α) value** | **Interpretation** | **Action** |
| --- | --- | --- |
| α = 1 | Perfect agreement | - Accept mapping outcomes for drawing conclusions - No disagreements to resolve |
| α ≥ 0.80 | Satisfactory level of agreement | - Accept mapping outcomes for drawing conclusions - Resolve disagreements |
| 0.67 ≤ α < 0.80 | Tentative agreement | - Interpret mapping outcomes with caution - Resolve disagreements |
| α < 0.67 | Poor agreement | - Re-examine mapping rules - Provide additional coder training - Resolve disagreements |
| α = 0 | Agreement at chance level | - Re-examine mapping rules - Provide additional coder training - Re-code all mappings - Resolve disagreements |
| α < 0 | Systematic disagreement | - Re-examine mapping rules - Provide additional coder training - Re-code all mappings - Resolve disagreements |

**Comment_R1#4:** Expand the limitations section, especially regarding restricted mapping scope:

The current limitations section mainly addresses the exclusion of “health emergency” concepts. However, the discussion could be expanded to better reflect other important methodological constraints. A more comprehensive limitations section would strengthen the interpretation of the eventual findings.

In particular, several additional limitations merit explicit discussion on following areas;

a) The mapping scope is restricted to the ICD-11 Foundation sections “External causes of morbidity or mortality” and the related extension codes under “Dimensions of external causes”. This restriction may exclude potentially relevant entities from other ICD-11_F sections that could capture aspects of emergency events, injury mechanisms, or contextual response settings.

**Response_R1#4a: Agree**. We added a limitation regarding the restriction to these two ICD-11_F sections, as follows:

We deliberately restricted the scope to the ICD-11_F sections "External causes of morbidity or mortality" and the related extension codes under "Dimensions of external causes," following a detailed review of the current ERC_T concepts against the ICD-11_F. However, other ICD-11_F sections may capture additional aspects of emergency events, injury mechanisms, or contextual response settings. Extending the ERC_T concepts to cover such entities lies beyond the aim of this registered report protocol and will be considered in future work.

b) The predefined one-to-one mapping procedure may oversimplify concepts that could be more accurately represented through multi-target or compositional mapping.

**Response_R1#4b: Agree**. We addressed this concern in our "Response_R1#1", which justifies the one-to-one mapping cardinality and acknowledges its limitations.

c) Possible coder subjectivity in semantic interpretation.

**Response_R1#4c: Agree.** We added a limitation regarding coder subjectivity in semantic interpretation, as follows:

There is also the possibility of coder subjectivity in semantic interpretation. We will mitigate this by applying predefined mapping decision rules and mapping examples. We will further diagnose coder subjectivity through inter-coder reliability analysis (Section 2.6.3) and resolve it through structured consensus discussions.

d) The generalizability of findings to broader emergency informatics contexts.

**Response_R1#4d: Agree**. We added a limitation regarding the generalizability of findings to broader emergency informatics contexts as follows:

The findings of this study are specific to the terminology mapping between ERC_T and ICD-11_F within the defined scope, and cannot be directly generalized to other emergency informatics contexts.

**Comment_R1#5:** Correct minor typographical and grammatical issues:

The manuscript is clearly written in standard academic English and is generally well organized, though minor typographical and grammatical corrections are recommended. (e.g., page 22, line 419, “identifiy” should be corrected to “identify”). Additionally, some long sentences in the Methods and Discussion sections may be divided into shorter sentences for improved readability.

**Response_R1#5: Agree**. We have corrected 'identifiy' to 'identify,' fixed other grammatical issues, and shortened long sentences for improved readability.

------------------------------------------------------

**Reviewer #2 (R2):**

I. **General comments**

-------------------------------------------------------

The study titled "Mapping the terminology of the early rescue chain to the Foundation of ICD-11: Registered report protocol" presents a well-structured approach to addressing the semantic interoperability challenges in the Early Rescue Chain (ERC). This study is a timely and relevant topic and aligns with WHO's emphasis on digital technologies as accelerators for health preparedness and response. The focus on ERC_T to ICD-11 foundation is innovative. The methodology is strong. The use of predefined mapping criteria, independent coders, and structured consensus discussions enhances the reliability and reproducibility of the study. There are also opportunities for the improvement of this process by developing algorithms to automate the mapping process. The methodology could be extended to other terminologies and classifications to further enhance semantic interoperability in health informatics.

The manuscript provides a significant contribution to the field of health informatics and emergency response systems. Its innovative methodology, rigorous approach, and potential impact on global health emergency preparedness make it a strong candidate for publication in PLOS ONE.

**Response:** We thank you for your positive assessment of our registered report protocol. We appreciate your suggestions to improve the mapping process by developing algorithms and extending the methodology to other terminologies and classifications. We have added these suggestions as future research directions at the end of Section 3.1 (Anticipated outcomes), as follows:

This registered report protocol provides a foundation for future research. Such research may include developing algorithms to automate the mapping process and extending the methodology to other terminologies and classifications in health informatics.
