# Supplementary material for: Mapping the terminology of the early rescue chain to the Foundation of ICD-11: Registered report protocol
Source: PLoS One. 2026 Jun 16;21(6):e0350733. doi: 10.1371/journal.pone.0350733 (PMC13271442; doi:10.1371/journal.pone.0350733)
Supplement: Peer review history

# Peer review: Decision Letter 0

**DOI:** 10.1371/journal.pone.0350733.r001

**Authors:** Pasyodun Koralage Buddhika Mahesh

15 Apr 2026

PONE-D-26-10568

Mapping the terminology of the early rescue chain to the Foundation of ICD-11: Registered report protocol

PLOS One

Dear Dr. Tegegne,

Thank you for submitting your manuscript to PLOS ONE. After careful consideration, we feel that it has merit but does not fully meet PLOS ONE’s publication criteria as it currently stands. Therefore, we invite you to submit a revised version of the manuscript that addresses the points raised during the review process.

Please submit your revised manuscript by May 30 2026 11:59PM. If you will need more time than this to complete your revisions, please reply to this message or contact the journal office at plosone@plos.org. When you're ready to submit your revision, log on to https://www.editorialmanager.com/pone/ and select the 'Submissions Needing Revision' folder to locate your manuscript file.

Please include the following items when submitting your revised manuscript:

- A letter that responds to each point raised by the academic editor and reviewer(s). You should upload this letter as a separate file labeled 'Response to Reviewers'.
- A marked-up copy of your manuscript that highlights changes made to the original version. You should upload this as a separate file labeled 'Revised Manuscript with Track Changes'.
- An unmarked version of your revised paper without tracked changes. You should upload this as a separate file labeled 'Manuscript'.

If you would like to make changes to your financial disclosure, please include your updated statement in your cover letter. Guidelines for resubmitting your figure files are available below the reviewer comments at the end of this letter.

If applicable, we recommend that you deposit your laboratory protocols in protocols.io to enhance the reproducibility of your results. Protocols.io assigns your protocol its own identifier (DOI) so that it can be cited independently in the future. For instructions see: https://journals.plos.org/plosone/s/submission-guidelines#loc-laboratory-protocols. Additionally, PLOS ONE offers an option for publishing peer-reviewed Lab Protocol articles, which describe protocols hosted on protocols.io. Read more information on sharing protocols at https://plos.org/protocols?utm_medium=editorial-email&utm_source=authorletters&utm_campaign=protocols.

As the corresponding author, your ORCID iD is verified in the submission system and will appear in the published article. PLOS supports the use of ORCID, and we encourage all coauthors to register for an ORCID iD and use it as well. Please encourage your coauthors to verify their ORCID iD within the submission system before final acceptance, as unverified ORCID iDs will not appear in the published article. *Only*  the individual author can complete the verification step; PLOS staff *cannot*  verify ORCID iDs on behalf of authors.

We look forward to receiving your revised manuscript.

Kind regards,

Pasyodun Koralage Buddhika Mahesh

Academic Editor

PLOS One

Journal Requirements:

When submitting your revision, we need you to address these additional requirements.

1. Please ensure that your manuscript meets PLOS ONE's style requirements, including those for file naming. The PLOS ONE style templates can be found at

https://journals.plos.org/plosone/s/file?id=wjVg/PLOSOne_formatting_sample_main_body.pdf and https://journals.plos.org/plosone/s/file?id=ba62/PLOSOne_formatting_sample_title_authors_affiliations.pdf

2. In your cover letter, please confirm that the research you have described in your manuscript, including participant recruitment, data collection, modification, or processing, has not started and will not start until after your paper has been accepted to the journal (assuming data need to be collected or participants recruited specifically for your study). In order to proceed with your submission, you must provide confirmation.

3. Please note that PLOS One has specific guidelines on code sharing for submissions in which author-generated code underpins the findings in the manuscript. In these cases, we expect all author-generated code to be made available without restrictions upon publication of the work. Please review our guidelines at https://journals.plos.org/plosone/s/materials-and-software-sharing#loc-sharing-code and ensure that your code is shared in a way that follows best practice and facilitates reproducibility and reuse.

If the reviewer comments include a recommendation to cite specific previously published works, please review and evaluate these publications to determine whether they are relevant and should be cited. There is no requirement to cite these works unless the editor has indicated otherwise.

Please review your reference list to ensure that it is complete and correct. If you have cited papers that have been retracted, please include the rationale for doing so in the manuscript text, or remove these references and replace them with relevant current references. Any changes to the reference list should be mentioned in the rebuttal letter that accompanies your revised manuscript. If you need to cite a retracted article, indicate the article’s retracted status in the References list and also include a citation and full reference for the retraction notice.

[Note: HTML markup is below. Please do not edit.]

Reviewers' comments:

Reviewer's Responses to Questions

**Comments to the Author**

1. Does the manuscript provide a valid rationale for the proposed study, with clearly identified and justified research questions?

The research question outlined is expected to address a valid academic problem or topic and contribute to the base of knowledge in the field.

Reviewer #1: Yes

Reviewer #2: Yes

**********

2. Is the protocol technically sound and planned in a manner that will lead to a meaningful outcome and allow testing the stated hypotheses?

The manuscript should describe the methods in sufficient detail to prevent undisclosed flexibility in the experimental procedure or analysis pipeline, including sufficient outcome-neutral conditions (e.g. necessary controls, absence of floor or ceiling effects) to test the proposed hypotheses and a statistical power analysis where applicable. As there may be aspects of the methodology and analysis which can only be refined once the work is undertaken, authors should outline potential assumptions and explicitly describe what aspects of the proposed analyses, if any, are exploratory.

Reviewer #1: Partly

Reviewer #2: Yes

**********

3. Is the methodology feasible and described in sufficient detail to allow the work to be replicable?

Reviewer #1: Yes

Reviewer #2: Yes

**********

4. Have the authors described where all data underlying the findings will be made available when the study is complete?

The PLOS Data policy requires authors to make all data underlying the findings described in their manuscript fully available without restriction, with rare exception, at the time of publication. The data should be provided as part of the manuscript or its supporting information, or deposited to a public repository. For example, in addition to summary statistics, the data points behind means, medians and variance measures should be available. If there are restrictions on publicly sharing data—e.g. participant privacy or use of data from a third party—those must be specified.

Reviewer #1: Yes

Reviewer #2: Yes

**********

5. Is the manuscript presented in an intelligible fashion and written in standard English?

*PLOS ONE*  does not copyedit accepted manuscripts, so the language in submitted articles must be clear, correct, and unambiguous. Any typographical or grammatical errors should be corrected at revision, so please note any specific errors here.

Reviewer #1: Yes

Reviewer #2: Yes

**********

6. Review Comments to the Author

Please use the space provided to explain your answers to the questions above and, if applicable, provide comments about issues authors must address before this protocol can be accepted for publication. You may also include additional comments for the author, including concerns about research or publication ethics.

You may also provide optional suggestions and comments to authors that they might find helpful in planning their study.

(Please upload your review as an attachment if it exceeds 20,000 characters)

Reviewer #1: This well-designed, timely registered report protocol addresses an important issue in emergency health informatics and semantic interoperability. The manuscript has several strengths, including strong academic rationale, use of internationally accepted standards, rigorous mapping methodology, independent coder-based validation, and appropriate reliability assessment. The planned study is likely to make a meaningful contribution to the interoperability of emergency response systems and the use of ICD-11 in automated health information exchange.

I recommend minor revisions to strengthen methodological transparency and reproducibility to the following points;

1. Provide stronger justification for the one-to-one mapping cardinality:

The protocol specifies a one-to-one mapping approach, whereby each ERC_T concept is mapped to a single ICD-11_F target concept. While this simplifies the mapping framework and may facilitate analysis, the rationale for restricting the study to this cardinality should be more explicitly justified.

In terminology mapping studies, especially when working with rich semantic resources such as the ICD-11 Foundation, concepts may sometimes be more accurately represented through one-to-many mappings, post-coordination, etc. Restricting the process to a single target concept may underestimate the true semantic alignment and could potentially inflate the number of “no match” or low-quality mappings. The authors are encouraged to provide a clear justification for this design choice and briefly discuss how this restriction may influence mapping outcomes.

2. Clarify the decision rules for ambiguous or partially overlapping concepts:

The protocol explains how to use HL7 FHIR ConceptMap relationships and ISO 21564 quality scores, however it should go into more depth about how to handle ambiguous ideas, partially overlapping concepts, and numerous equally plausible targets. I suggest including one or two additional worked examples of “related-to” mappings, particularly to illustrate how coders should distinguish between quality scores 2 and 3 in borderline cases where semantic overlap is not immediately obvious. Providing clearer operational rules or additional worked examples would improve reproducibility and reduce subjective variation between coders.

3. Include predefined thresholds for Krippendorff’s alpha interpretation:

The use of Krippendorff’s alpha is appropriate and strengthens the methodological rigor of the study. However, the protocol would benefit from including predefined thresholds for interpretation. Including such thresholds is particularly important in a registered report protocol, as it reduces analytical flexibility and improves transparency in how intercoder reliability will be interpreted.

4. Expand the limitations section, especially regarding restricted mapping scope:

The current limitations section mainly addresses the exclusion of “health emergency” concepts. However, the discussion could be expanded to better reflect other important methodological constraints.

In particular, several additional limitations merit explicit discussion on following areas;

a) The mapping scope is restricted to the ICD-11 Foundation sections “External causes of morbidity or mortality” and the related extension codes under “Dimensions of external causes”. This restriction may exclude potentially relevant entities from other ICD-11_F sections that could capture aspects of emergency events, injury mechanisms, or contextual response settings.

b) The predefined one-to-one mapping procedure may oversimplify concepts that could be more accurately represented through multi-target or compositional mapping.

c) Possible coder subjectivity in semantic interpretation.

d) The generalizability of findings to broader emergency informatics contexts.

A more comprehensive limitations section would strengthen the interpretation of the eventual findings.

5. Correct minor typographical and grammatical issues:

The manuscript is clearly written in standard academic English and is generally well organized, though minor typographical and grammatical corrections are recommended. (e.g., page 22, line 419, “identifiy” should be corrected to “identify”). Additionally, some long sentences in the Methods and Discussion sections may be divided into shorter sentences for improved readability.

Reviewer #2: The study titled "Mapping the terminology of the early rescue chain to the Foundation of ICD-11: Registered report protocol" presents a well-structured approach to addressing the semantic interoperability challenges in the Early Rescue Chain (ERC). This study is a timely and relevant topic and aligns with WHO's emphasis on digital technologies as accelerators for health preparedness and response. The focus on ERC_T to ICD-11 foundation is innovative. The methodology is strong. The use of predefined mapping criteria, independent coders, and structured consensus discussions enhances the reliability and reproducibility of the study. There are also opportunities for the improvement of this process by developing algorithms to automate the mapping process. The methodology could be extended to other terminologies and classifications to further enhance semantic interoperability in health informatics.

The manuscript provides a significant contribution to the field of health informatics and emergency response systems. Its innovative methodology, rigorous approach, and potential impact on global health emergency preparedness make it a strong candidate for publication in PLOS ONE.

**********

7. PLOS authors have the option to publish the peer review history of their article (what does this mean?). If published, this will include your full peer review and any attached files.

If you choose “no”, your identity will remain anonymous but your review may still be made public.

**Do you want your identity to be public for this peer review?**  For information about this choice, including consent withdrawal, please see our Privacy Policy.

Reviewer #1: **Yes:**  Viraj Udayanga

Reviewer #2: **Yes:**  U S H GAMAGE

**********

[NOTE: If reviewer comments were submitted as an attachment file, they will be attached to this email and accessible via the submission site. Please log into your account, locate the manuscript record, and check for the action link "View Attachments". If this link does not appear, there are no attachment files.]

To ensure your figures meet our technical requirements, please review our figure guidelines: https://journals.plos.org/plosone/s/figures

You may also use PLOS’s free figure tool, NAAS, to help you prepare publication quality figures: https://journals.plos.org/plosone/s/figures#loc-tools-for-figure-preparation.

NAAS will assess whether your figures meet our technical requirements by comparing each figure against our figure specifications.

# Author comment: Author response to Decision Letter 1

**DOI:** 10.1371/journal.pone.0350733.r002

4 May 2026

Response letter for "[PONE-D-26-10568]: Mapping the terminology of the early rescue chain to the Foundation of ICD-11: Registered report protocol."

Dear Editor and Reviewers,

We thank the editorial team and reviewers for their time, expertise, and constructive feedback.

We have prepared a point-by-point response addressing each journal requirement and reviewer suggestion. For each point, we first restate the comment, followed by our detailed response. Amendments are clearly marked in the revised manuscript using track changes.

In addition, we hereby confirm that the research we have described in our manuscript, including participant recruitment, data collection, modification, or processing, has not started and will not start until after our paper has been accepted to the journal PLOS One. Furthermore, for our study, we do not need to collect data or recruit participants.

Please let us know if any further changes or clarifications are needed.

Best regards,

The Authors

------------------------------------------------------

Journal Requirements (JR)

-------------------------------------------------------

When submitting your revision, we need you to address these additional requirements.

Comment_JR#1: Please ensure that your manuscript meets PLOS ONE's style requirements, including those for file naming. The PLOS ONE style templates can be found at https://journals.plos.org/plosone/s/file?id=wjVg/PLOSOne_formatting_sample_main_body.pdf and https://journals.plos.org/plosone/s/file?id=ba62/PLOSOne_formatting_sample_title_authors_affiliations.pdf

Response_JR#1: We confirm that we have prepared the manuscript in accordance with PLOS ONE's style requirements, including file naming conventions.

Comment_JR#2: In your cover letter, please confirm that the research you have described in your manuscript, including participant recruitment, data collection, modification, or processing, has not started and will not start until after your paper has been accepted to the journal (assuming data need to be collected or participants recruited specifically for your study). In order to proceed with your submission, you must provide confirmation.

Response_JR#2: We have revised the confirmation statement in our cover letter to address this requirement, as follows:

We hereby confirm that the research we have described in our manuscript, including participant recruitment, data collection, modification, or processing, has not started and will not start until after our paper has been accepted to the journal PLOS One. Furthermore, for our study, we do not need to collect data or recruit participants.

Comment_JR#3: Please note that PLOS One has specific guidelines on code sharing for submissions in which author-generated code underpins the findings in the manuscript. In these cases, we expect all author-generated code to be made available without restrictions upon publication of the work. Please review our guidelines at https://journals.plos.org/plosone/s/materials-and-software-sharing#loc-sharing-code and ensure that your code is shared in a way that follows best practice and facilitates reproducibility and reuse.

Response_JR#3: We confirm that no author-generated code has been developed at this stage. The planned analyses are descriptive in nature and will be performed using standard statistical software.

Comment_JR#4: If the reviewer comments include a recommendation to cite specific previously published works, please review and evaluate these publications to determine whether they are relevant and should be cited. There is no requirement to cite these works unless the editor has indicated otherwise.

Response_JR#4: As the reviewers did not recommend specific citations, no additional references have been included.

Comment_JR#5: Please review your reference list to ensure that it is complete and correct. If you have cited papers that have been retracted, please include the rationale for doing so in the manuscript text, or remove these references and replace them with relevant current references. Any changes to the reference list should be mentioned in the rebuttal letter that accompanies your revised manuscript. If you need to cite a retracted article, indicate the article’s retracted status in the References list and also include a citation and full reference for the retraction notice.

Response_JR#5: We have carefully reviewed our reference list to ensure completeness and accuracy. We do not cite any retracted articles in the manuscript.

------------------------------------------------------

Reviewer #1 (R1)

I. General comments

-------------------------------------------------------

This well-designed, timely registered report protocol addresses an important issue in emergency health informatics and semantic interoperability. The manuscript has several strengths, including strong academic rationale, use of internationally accepted standards, rigorous mapping methodology, independent coder-based validation, and appropriate reliability assessment. The planned study is likely to make a meaningful contribution to the interoperability of emergency response systems and the use of ICD-11 in automated health information exchange.

Response: We appreciate your positive assessment of our registered report protocol. We have thoroughly addressed all subsequent comments below to further enhance the manuscript.

II. Specific comments

-------------------------------------------------------

Comment_R1#1: Provide stronger justification for the one-to-one mapping cardinality.

The protocol specifies a one-to-one mapping approach, whereby each ERC_T concept is mapped to a single ICD-11_F target concept. While this simplifies the mapping framework and may facilitate analysis, the rationale for restricting the study to this cardinality should be more explicitly justified.

In terminology mapping studies, especially when working with rich semantic resources such as the ICD-11 Foundation, concepts may sometimes be more accurately represented through one-to-many mappings, post-coordination, etc. Restricting the process to a single target concept may underestimate the true semantic alignment and could potentially inflate the number of “no match” or low-quality mappings. The authors are encouraged to provide a clear justification for this design choice and briefly discuss how this restriction may influence mapping outcomes.

Response_R1#1: Agree. We provided an explicit justification for the one-to-one cardinality at the end of Section 2.4 (Scope and eligibility criteria), which reads as follows:

We follow a one-to-one mapping cardinality. This is because ERC_T is a hierarchical terminology of predefined concepts (e.g., a specific event, location, transport type, or type of room) rather than a collection of pre-coordinated composite terms. The target, ICD-11_F, is a terminology in which a URI uniquely identifies each entity. Therefore, single-target semantic equivalence is the appropriate approach for assessing semantic alignment between the two terminologies. Compositional cardinality scenarios (one-to-many, many-to-one, or many-to-many) lie outside the scope of this terminology mapping, as they pertain to the combined use of terminologies in real-world coding, such as encoding a complete event record.

In addition, we have acknowledged how this restriction may influence mapping outcomes in the Anticipated Limitations section, which reads as follows:

Although the source terminology is a nomenclature, ICD-11_F is semantically rich and may contain several plausible target entities for a single source concept. In these cases, the coder must select the most appropriate single target, a subjective process that may degrade mapping quality.

We also note that our mitigation measures to manage coder subjectivity are described in "Response_R1#4c".

Comment_R1#2: Clarify the decision rules for ambiguous or partially overlapping concepts:

The protocol explains how to use HL7 FHIR ConceptMap relationships and ISO 21564 quality scores, however it should go into more depth about how to handle ambiguous ideas, partially overlapping concepts, and numerous equally plausible targets. I suggest including one or two additional worked examples of “related-to” mappings, particularly to illustrate how coders should distinguish between quality scores 2 and 3 in borderline cases where semantic overlap is not immediately obvious. Providing clearer operational rules or additional worked examples would improve reproducibility and reduce subjective variation between coders.

Response_R1#2: Agree. We clarified our decision rules by adding two examples that illustrate how coders should distinguish between quality scores "2" and "3" for "related-to" mappings, as follows:

Example 1 (Quality score 2: "Related to" with a well-defined intersection):

The ERC_T concept "Residential area," defined as "Outdoor land area used for daily living activities," maps to the ICD-11_F entity "Home." The semantic relationship between the two is "Related to," as neither concept fully subsumes the other: ERC_T concept covers all outdoor residential areas, including those that extend beyond homes (e.g., parks, sports and entertainment areas, squares, and marketplaces), whereas ICD-11_F's "Home" is limited to a single dwelling and its indoor spaces. The two concepts share a well-defined intersection: the outdoor spaces directly attached to a single dwelling (e.g., garden, roof, backyard, garage). The resulting quality score is "2" (the source is partially included in the target).

Example 2 (Quality score 3: "Related to" without a well-defined intersection):

The ERC_T concept "Diver-assisted device," defined as "Underwater transport devices that directly augment a diver's capabilities," maps to the ICD-11_F entity "Underwater diving equipment." The semantic relationship is "Related to" because both refer to equipment used by divers underwater. However, there is no well-defined intersection, as the ERC_T concept refers to devices that actively assist a diver's movement (e.g., propulsion devices, assist exoskeletons), whereas ICD-11_F refers to the passive gear divers wear underwater (e.g., aqualung, wetsuit, mask, fins). This results in a quality score of "3" (Source is mapped; however, there are many options of possible concepts and overlaps. The map produced is the best comparison rather than an actual correspondence. Such a map requires significant manual input to create and maintain, and should be used carefully).

Comment_R1#3: Include predefined thresholds for Krippendorff’s alpha interpretation:

The use of Krippendorff’s alpha is appropriate and strengthens the methodological rigor of the study. However, the protocol would benefit from including predefined thresholds for interpretation. Including such thresholds is particularly important in a registered report protocol, as it reduces analytical flexibility and improves transparency in how intercoder reliability will be interpreted.

Response_R1#3: Agree. We added a new table (Table 6) in Section 2.6.3 (Quality assurance), presenting predefined thresholds for Krippendorff's alpha (α) interpretation, adapted from Krippendorff [35] and Marzi et al. [36], along with the recommended action at each threshold. The added section reads as follows:

We will calculate the percentage agreement of target entity selection and evaluate intercoder reliability using Krippendorff's alpha (α) [35]. We will interpret inter-coder reliability using the predefined thresholds (Table 6), adapted from Krippendorff [35] and Marzi et al. [36]. In cases of disagreement, coders will resolve them through structured consensus discussions. If they cannot reach a consensus, they will refer the case to the WHO-FIC expert for final decision.

-----------[Please refer to Table 6 in the attached "Response to Reviewers" document for the formatted version.]---------

Comment_R1#4: Expand the limitations section, especially regarding restricted mapping scope:

The current limitations section mainly addresses the exclusion of “health emergency” concepts. However, the discussion could be expanded to better reflect other important methodological constraints. A more comprehensive limitations section would strengthen the interpretation of the eventual findings.

In particular, several additional limitations merit explicit discussion on following areas;

a) The mapping scope is restricted to the ICD-11 Foundation sections “External causes of morbidity or mortality” and the related extension codes under “Dimensions of external causes”. This restriction may exclude potentially relevant entities from other ICD-11_F sections that could capture aspects of emergency events, injury mechanisms, or contextual response settings.

Response_R1#4a: Agree. We added a limitation regarding the restriction to these two ICD-11_F sections, as follows:

We deliberately restricted the scope to the ICD-11_F sections "External causes of morbidity or mortality" and the related extension codes under "Dimensions of external causes," following a detailed review of the current ERC_T concepts against the ICD-11_F. However, other ICD-11_F sections may capture additional aspects of emergency events, injury mechanisms, or contextual response settings. Extending the ERC_T concepts to cover such entities lies beyond the aim of this registered report protocol and will be considered in future work.

b) The predefined one-to-one mapping procedure may oversimplify concepts that could be more accurately represented through multi-target or compositional mapping.

Response_R1#4b: Agree. We addressed this concern in our "Response_R1#1", which justifies the one-to-one mapping cardinality and acknowledges its limitations.

c) Possible coder subjectivity in semantic interpretation.

Response_R1#4c: Agree. We added a limitation regarding coder subjectivity in semantic interpretation, as follows:

There is also the possibility of coder subjectivity in semantic interpretation. We will mitigate this by applying predefined mapping decision rules and mapping examples. We will further diagnose coder subjectivity through inter-coder reliability analysis (Section 2.6.3) and resolve it through structured consensus discussions.

d) The generalizability of findings to broader emergency informatics contexts.

Response_R1#4d: Agree. We added a limitation regarding the generalizability of findings to broader emergency informatics contexts as follows:

The findings of this study are specific to the terminology mapping between ERC_T and ICD-11_F within the defined scope, and cannot be directly generalized to other emergency informatics contexts.

Comment_R1#5: Correct minor typographical and grammatical issues:

The manuscript is clearly written in standard academic English and is generally well organized, though minor typographical and grammatical corrections are recommended. (e.g., page 22, line 419, “identifiy” should be corrected to “identify”). Additionally, some long sentences in the Methods and Discussion sections may be divided into shorter sentences for improved readability.

Response_R1#5: Agree. We have corrected 'identifiy' to 'identify,' fixed other grammatical issues, and shortened long sentences for improved readability.

------------------------------------------------------

Reviewer #2 (R2):

I. General comments

-------------------------------------------------------

The study titled "Mapping the terminology of the early rescue chain to the Foundation of ICD-11: Registered report protocol" presents a well-structured approach to addressing the semantic interoperability challenges in the Early Rescue Chain (ERC). This study is a timely and relevant topic and aligns with WHO's emphasis on digital technologies as accelerators for health preparedness and response. The focus on ERC_T to ICD-11 foundation is innovative. The methodology is strong. The use of predefined mapping criteria, independent coders, and structured consensus discussions enhances the reliability and reproducibility of the study. There are also opportunities for the improvement of this process by developing algorithms to automate the mapping process. The methodology could be extended to other terminologies and classifications to further enhance semantic interoperability in health informatics.

The ma

Attachment Submitted filename: Response to Reviewers.docx

# Peer review: Decision Letter 1

**DOI:** 10.1371/journal.pone.0350733.r003

**Authors:** Pasyodun Koralage Buddhika Mahesh

19 May 2026

Mapping the terminology of the early rescue chain to the Foundation of ICD-11: Registered report protocol

PONE-D-26-10568R1

Dear Dr. Tegegne,

We’re pleased to inform you that your manuscript has been judged scientifically suitable for publication and will be formally accepted for publication once it meets all outstanding technical requirements.

Within one week, you’ll receive an e-mail detailing the required amendments. When these have been addressed, you’ll receive a formal acceptance letter and your manuscript will be scheduled for publication.

An invoice will be generated when your article is formally accepted. Please note, if your institution has a publishing partnership with PLOS and your article meets the relevant criteria, all or part of your publication costs will be covered. Please make sure your user information is up-to-date by logging into Editorial Manager at Editorial Manager® and clicking the ‘Update My Information' link at the top of the page. For questions related to billing, please contact billing support.

If your institution or institutions have a press office, please notify them about your upcoming paper to help maximize its impact. If they’ll be preparing press materials, please inform our press team as soon as possible -- no later than 48 hours after receiving the formal acceptance. Your manuscript will remain under strict press embargo until 2 pm Eastern Time on the date of publication. For more information, please contact onepress@plos.org.

Kind regards,

Pasyodun Koralage Buddhika Mahesh

Academic Editor

PLOS One

Additional Editor Comments (optional):

Reviewers' comments:

Reviewer's Responses to Questions

**Comments to the Author**

1. Does the manuscript provide a valid rationale for the proposed study, with clearly identified and justified research questions?

The research question outlined is expected to address a valid academic problem or topic and contribute to the base of knowledge in the field.

Reviewer #1: Yes

Reviewer #2: Yes

**********

2. Is the protocol technically sound and planned in a manner that will lead to a meaningful outcome and allow testing the stated hypotheses?

The manuscript should describe the methods in sufficient detail to prevent undisclosed flexibility in the experimental procedure or analysis pipeline, including sufficient outcome-neutral conditions (e.g. necessary controls, absence of floor or ceiling effects) to test the proposed hypotheses and a statistical power analysis where applicable. As there may be aspects of the methodology and analysis which can only be refined once the work is undertaken, authors should outline potential assumptions and explicitly describe what aspects of the proposed analyses, if any, are exploratory.

Reviewer #1: Yes

Reviewer #2: Yes

**********

3. Is the methodology feasible and described in sufficient detail to allow the work to be replicable?

Reviewer #1: Yes

Reviewer #2: Yes

**********

4. Have the authors described where all data underlying the findings will be made available when the study is complete?

The PLOS Data policy requires authors to make all data underlying the findings described in their manuscript fully available without restriction, with rare exception, at the time of publication. The data should be provided as part of the manuscript or its supporting information, or deposited to a public repository. For example, in addition to summary statistics, the data points behind means, medians and variance measures should be available. If there are restrictions on publicly sharing data—e.g. participant privacy or use of data from a third party—those must be specified.

Reviewer #1: Yes

Reviewer #2: Yes

**********

5. Is the manuscript presented in an intelligible fashion and written in standard English?

*PLOS ONE*  does not copyedit accepted manuscripts, so the language in submitted articles must be clear, correct, and unambiguous. Any typographical or grammatical errors should be corrected at revision, so please note any specific errors here.

Reviewer #1: Yes

Reviewer #2: Yes

**********

6. Review Comments to the Author

Please use the space provided to explain your answers to the questions above and, if applicable, provide comments about issues authors must address before this protocol can be accepted for publication. You may also include additional comments for the author, including concerns about research or publication ethics.

You may also provide optional suggestions and comments to authors that they might find helpful in planning their study.

(Please upload your review as an attachment if it exceeds 20,000 characters)

Reviewer #1: The authors have sufficiently addressed the reviewer comments from the previous round of review and have significantly improved the manuscript. The revised protocol is a scientifically sound and methodologically rigorous approach to the terminology mapping between the ERC terminology and the ICD-11 Foundation. This study aims at an important interoperability challenge in emergency informatics and has potential relevance in future standardization efforts in digital health systems. Therefore, in my opinion overall the manuscript is well-structured, technically appropriate for a registered report protocol and suitable for publication in PLOS ONE.

Reviewer #2: The authors have successfully addressed the reviewer comments. They have accepted my suggestion to improve the process by developing algorithms to automate the mapping process in future research. The manuscript provides a significant contribution to the field of health informatics and emergency response systems. Its innovative methodology, rigorous approach, and potential impact on global health emergency preparedness make it a strong candidate for publication in PLOS ONE.

**********

7. PLOS authors have the option to publish the peer review history of their article (what does this mean?). If published, this will include your full peer review and any attached files.

If you choose “no”, your identity will remain anonymous but your review may still be made public.

**Do you want your identity to be public for this peer review?**  For information about this choice, including consent withdrawal, please see our Privacy Policy.

Reviewer #1: **Yes:**  Viraj Udayanga

Reviewer #2: **Yes:**  UDAYA SAMANTHILAKA HATTOTUWA GAMAGE

**********

# Editor report: Acceptance letter

**DOI:** 10.1371/journal.pone.0350733.r004

**Authors:** Pasyodun Koralage Buddhika Mahesh

PONE-D-26-10568R1

PLOS One

Dear Dr. Tegegne,

I'm pleased to inform you that your manuscript has been deemed suitable for publication in PLOS One. Congratulations! Your manuscript is now being handed over to our production team.

At this stage, our production department will prepare your paper for publication. This includes ensuring the following:

* All references, tables, and figures are properly cited

* All relevant supporting information is included in the manuscript submission,

* There are no issues that prevent the paper from being properly typeset

You will receive further instructions from the production team, including instructions on how to review your proof when it is ready. Please keep in mind that we are working through a large volume of accepted articles, so please give us a few days to review your paper and let you know the next and final steps.

Lastly, if your institution or institutions have a press office, please let them know about your upcoming paper now to help maximize its impact. If they'll be preparing press materials, please inform our press team within the next 48 hours. Your manuscript will remain under strict press embargo until 2 pm Eastern Time on the date of publication. For more information, please contact onepress@plos.org.

You will receive an invoice from PLOS for your publication fee after your manuscript has reached the completed accept phase. If you receive an email requesting payment before acceptance or for any other service, this may be a phishing scheme. Learn how to identify phishing emails and protect your accounts at https://explore.plos.org/phishing.

If we can help with anything else, please email us at customercare@plos.org.

Thank you for submitting your work to PLOS ONE and supporting open access.

Kind regards,

PLOS ONE Editorial Office Staff

on behalf of

Dr. Pasyodun Koralage Buddhika Mahesh

Academic Editor

PLOS One
